# Green one-pot synthesis of recyclable cation-disordered Li_3_VO_4_ below 40 °C for high-rate anode materials

**DOI:** 10.1039/d6ra01593j

**Published:** 2026-03-18

**Authors:** Tatsuya Kondo, Sota Kawaguchi, Naoto Takeshima, Shuto Igari, Satoyuki Tatsumi, Kaori Akiyama, Kenji Machida, Sekihiro Takeda

**Affiliations:** a Basic Research Center, Nippon Chemi-Con Corporation KSP R&D C1025, 3-2-1 Sakado, Takatsu-ku, Kawasaki-shi Kanagawa 213-0012 Japan chem.edu.tkondo@gmail.com +81-44-379-6885 +81-44-379-6881; b Basic Research Center, Nippon Chemi-Con Corporation Takahagi Ibaraki 318-8505 Japan

## Abstract

To advance a sustainable society, increasing attention has been directed toward the development of high-performance materials and environmentally responsible synthetic methodologies. Cation-disordered materials have attracted attention across various fields because of their exceptional performance. However, their synthesis requires high-temperature calcination or after-treatment of cation-ordered precursors, resulting in high energy consumption levels, extended processing times, and increased costs, which hinder commercialization. Recently, low-temperature solution-based reactions enabling the synthesis of cation-disordered structures *via* instantaneous crystallization using spray dryers have been reported; however, technical insights into these processes remain unclear due to very few reports compared with those for solid-state reactions. In this study, we demonstrate low-temperature one-pot synthesis (<40 °C) of cation-disordered Li_3_VO_4_, a high-performance anode material, from a solution reaction *via* a combination of a rotary evaporator and a vacuum dryer, which are standard laboratory apparatuses, under strictly controlled conditions. This cation-disordered Li_3_VO_4_ is identified as a kinetically stabilized metastable phase and undergoes a moisture-induced phase transition to the thermodynamically stable β-phase, which results in inferior performance. Moreover, it can be dissolved in water and reverted to its precursor ions, indicating its recyclability. This methodology, in which a kinetically stabilized crystal phase is synthesized *via* evaporation-driven crystallization, supports the development of innovative materials that simultaneously achieve green chemistry and high performance.

## Abstract 2

The methodology supports the development of innovative materials that simultaneously achieve green chemistry and high performance.

## Graphical abstract

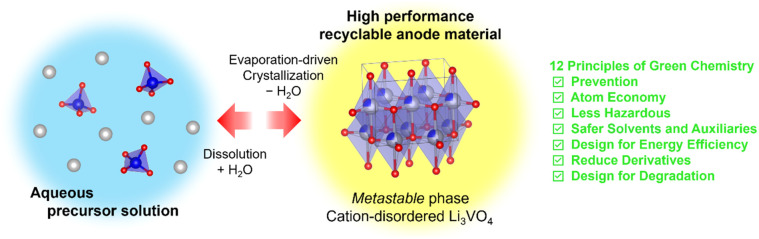

## Introduction

As society advances toward sustainability, there is a growing emphasis not only on designing high-performance materials but also on developing synthetic methodologies with minimal environmental impact. Even materials with outstanding functionality cannot be considered truly sustainable if their production involves excessive energy consumption. Therefore, establishing synthetic strategies that combine high performance with environmental sustainability remains a key objective in modern materials science.

Crystal structure plays a pivotal role in determining device performance, thus rendering its control crucial.^1,2^ In recent years, high-entropy crystal structures with cation disorder have attracted attention in various applications, including batteries,^3–5^ solid electrolytes,^6–8^ and semiconductors (such as thermoelectric^9,10^ and optoelectronic^11,12^), because of their potential to increase device performance. Particularly in lithium-ion batteries, the use of cation-disordered lithium transition metal oxides could improve electrochemical properties, such as increasing the capacity and voltage.^13–15^ For instance, compared with conventional commercialized cathode materials such as NCM (Lithium nickel cobalt manganese oxides) and LFP (LiFePO_4_), lithium-excess cation-disordered rock salt cathode materials offer higher energy density. Lithium-excess cation-disordered rock salt-type cathode materials have been reported to contain a wide range of variations, thereby incorporating not only Ni, Co, Mn, and Fe but also various other transition metals, such as Ti,^16–18^ V,^19–21^ Cr,^22^ Zr,^18^ Nb,^20,21,23^ Mo,^16,17,22^ and Ta.^24^ Among these elements, V can be used not only as a cathode^25–27^ material but also as an anode material because of its wide range of reaction potentials, which is based on its multiple oxidation states. Cation-disordered anode materials such as rock salt-type Li_3_V_2_O_5_ ^28^ and wurtzite-type Li_3_VO_4_ ^29–31^ are promising new anodes because of their high capacity, high-rate capabilities, and suitable potential to prevent the formation of lithium dendrites. Li_3_VO_4_ (LVO) is a promising insertion-type anode material,^32^ with a high theoretical capacity (394 mAh g^−1^, Li_3_V^5+^O_4_ + 2Li^+^ + 2e^−^ → Li_5_V^3+^O_4_) and a safe operating voltage range (0.4–1.3 V *vs.* Li^+^/Li). Although cation-ordered β-phase LVO, featuring an ordered arrangement of LiO_4_ and VO_4_ tetrahedra at room temperature, has been extensively reported as a lithium-ion battery anode,^33–45^ its superior potential as an anode material is limited by its low electronic conductivity (10^−10^ S cm^−1^).^42,43^ Naoi's group reported that cation-disordered LVO, with a lithium diffusion coefficient^30,31^ that is more than 100 times greater than that of β-phase LVO, demonstrates a remarkable rate performance^29,31^ and a capacity retention of greater than 50% at 20 A g^−1^. Despite these advantages, synthesis techniques for cation-disordered LVO remain challenging.

Lithium-excess cation-disordered rock salt cathode materials are generally synthesized *via* solid-state reactions, which involve mixing raw materials followed by high-temperature calcination. Other techniques, such as mechanochemical treatments with high-energy planetary ball milling^21^ or electrochemical treatments inducing disordered structures by repeated lithium-ion insertion/extraction,^22^ have been proposed. However, in the case of LVO, the direct synthesis of cation-disordered structures through calcination is restricted because of the formation of the thermodynamically stable γ-phase at high temperatures.^46–49^ Cation-disordered LVO has been synthesized through posttreatments for cation-ordered β-phase LVO, such as electrochemical (cycling between 2.5–0.1 V for 20 cycles)^30^ or mechanochemical (ball milling for 36 hours).^31^ However, these treatments require high energy consumption levels, extended processing times, and increased costs, making them impractical for large-scale commercialization (SI Table 1). Therefore, developing a novel synthesis technique capable of controlling the crystal phase of LVO while being energy efficient, eco-friendly, and cost effective for commercialization is essential.

The low-temperature synthesis methods for LVO with low energy consumption are summarized in Table 1. Conventional solid-state synthesis requires high temperatures above 600 °C for the reaction between lithium carbonate and vanadium oxide.^32^ In comparison, solution-based methods such as solvothermal^37^ or hydrothermal^38^ and precipitation^39^ methods have been reported to synthesize LVO at low temperatures without calcination. In particular, combining Li^+^ with VO_4_^3−^, a structural component of the LVO crystal structure generated *via* high-pH dissolution of NH_4_VO_3_,^38,39^ is a rational approach. Among previous reports, the humidity-assisted solid-state method at 80 °C represents the lowest-temperature synthesis method, in which LVO is formed through reactions of the raw material mixture inside water droplets.^45^ This method involves low energy consumption, is eco-friendly and does not involve the use of byproducts. Although these efforts have resulted in low-temperature, eco-friendly reactions, the resulting LVO shows primarily the β-phase, whose rate performance is inferior to that of the cation-disordered structure. For LVO design strategies, the crystal phase is a key factor contributing to high-rate performance, and the use of VO_4_^3−^ is critical for eco-friendly reactions. Recently, Naoi's group demonstrated the direct synthesis of cation-disordered LVO at 90 °C from an aqueous solution of LiOH and V_2_O_5_ through instantaneous crystallization *via* a spray-drying technique without passing *via* β-phase LVO.^50^ A balance between excellent electrochemical performance and eco-friendly reactions has been achieved, and a spray dryer is also suitable for industrial applications, as it is highly scalable and has an extensive history of successful implementation in industry. However, very few reports on the synthesis of cation-disordered materials through solution-based reactions, including the present reaction, exist, and technical insights regarding the synthesis remain insufficient compared with those for solid-state reactions. For example, many cation-disordered oxide materials synthesized *via* solid-state routes do not require any quenching-type processes to stabilize disordering, suggesting that ultrafast drying alone cannot universally explain the emergence of metastable disordered phases.

**Table 1:** Low-temperature LVO synthesis methods

Methodology	Source			Temperature^a^/°C	Energy cost^b^/J g^−1^	Reference
	Li	V	Solvent			
Solid-state	Li_2_CO_3_	V_2_O_5_	—	900	875	32
Solvothermal	Li_2_CO_3_	V_2_O_5_	H_2_O + acetone	300	853	37
Hydrothermal	LiOH (excess)	NH_4_VO_3_	H_2_O	180	651	38
Precipitation	LiOH (excess)	NH_4_VO_3_	Ethylene glycol	120	228	39
Humidity-assisted solid-state	LiOH	V_2_O_5_	H_2_O (droplet)	80	231	45
Spray-drying	LiOH	V_2_O_5_	H_2_O	90	65	50
**This work**	**LiOH**	**V**_**2**_ **O**_**5**_	**H**_**2**_ **O**	**40**	**63**	

a Temperature: the temperature refers to the reaction temperature used for the synthesis.

b Energy cost: the energy cost refers to the energy necessary to raise the reaction environment to the reaction temperature from room temperature (25 °C); this evaluation does not take into account factors such as the power consumption of the apparatus employed for the synthesis. The calculation formula is given below: (energy cost) = (specific heat capacity of the reaction environment) × (temperature difference between the reaction temperature and room temperature). Herein, the specific heat capacities (J g^−1^ K^−1^) used in the calculation are as follows: air: 1.0; H_2_O + acetone: 3.1; H_2_O: 4.2; and ethylene glycol: 2.4.

In this study, we demonstrate green one-pot synthesis of cation-disordered LVO below 40 °C with high-rate performance through a low-temperature solution reaction with strictly controlled drying processes using a rotary evaporator—which offers significantly slower drying than spray-drying—and a vacuum dryer, which are standard laboratory apparatuses. We experimentally and theoretically reveal that this cation-disordered structure is a kinetically stabilized metastable phase, and this phase readily transitions to the thermodynamically stable β-phase under catalytic action by moisture. This finding clarifies the key factors in the drying process required to obtain the metastable phase, highlighting the critical role of moisture control. In recent years, recycling technologies for lithium-ion batteries have been actively studied,^51,52^ and by exploiting the water solubility of LVO, facile resource recovery is anticipated, making it a promising recyclable and high-performance next-generation anode material.

## Results and discussion

### Preparation of cation-disordered LVO

**Fig. 1:**
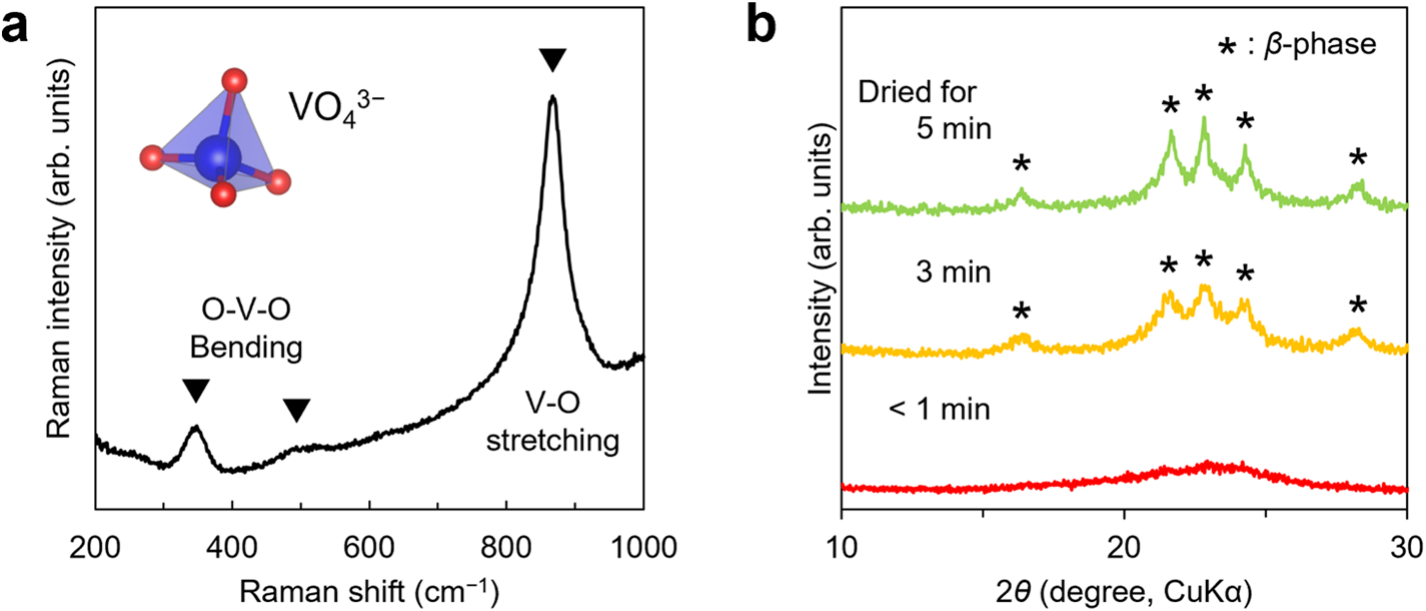
Preparation of cation-disordered LVO. (a) Raman spectra of the aqueous LVO precursor solution in the range of 200 to 1000 cm^−1^. (b) XRD patterns of the obtained powder after drying for different times in an evaporator.

We first optimized the synthesis conditions for preparing cation-disordered LVO, particularly the drying process. The formation of VO_4_^3−^ was confirmed by obtaining the Raman spectrum of the colourless and transparent LVO aqueous precursor solution obtained by dissolving LiOH and V_2_O_5_ in water at room temperature (Fig. 1a and S1). The observed peaks included those at 867 cm^−1^ from the V–O stretching vibration, 349 cm^−1^ and 497 cm^−1^ from the O–V–O bending vibration of the tetrahedral VO_4_^3−^ anion,^53,54^ and 1630 cm^−1^ and 2900–3800 cm^−1^ from H_2_O.^55^ According to the Pourbaix diagram, vanadium species can form tetrahedral orthovanadate anions in high-pH regions, while the formation of VO_4_^3−^ from V_2_O_5_ has also been confirmed.^56^ The aqueous precursor solution contains the components of LVO, since LVO exhibits a crystal structure comprising stacked tetrahedral LiO_4_ and VO_4_, with LiO_4_ formed by sharing the oxygen atoms of four VO_4_ units with Li^+^. When the LVO aqueous precursor solution was concentrated using an evaporator at 40 °C, a white powder suddenly appeared as the water completely evaporated. The X-ray diffraction (XRD) pattern of the resulting white powder indicated that it was β-phase LVO (space group: *Pmn*2_1_) (SI Fig. 2). The powder was dried in the rotary evaporator (<1000 Pa) for one hour after precipitation. However, owing to the significant amount of adsorbed water, further vacuum drying (<0.67 Pa) was performed to reduce the water content (SI Fig. 3). In those drying processes, reducing the drying time in the evaporator after precipitation caused the low-angle peaks (2*θ* < 30°) to gradually broaden, and when the drying time was less than one minute, the five peaks originating from the β-phase disappeared, yielding a single halo pattern (Fig. 1b).

### Crystal structure and physical properties

**Fig. 2:**
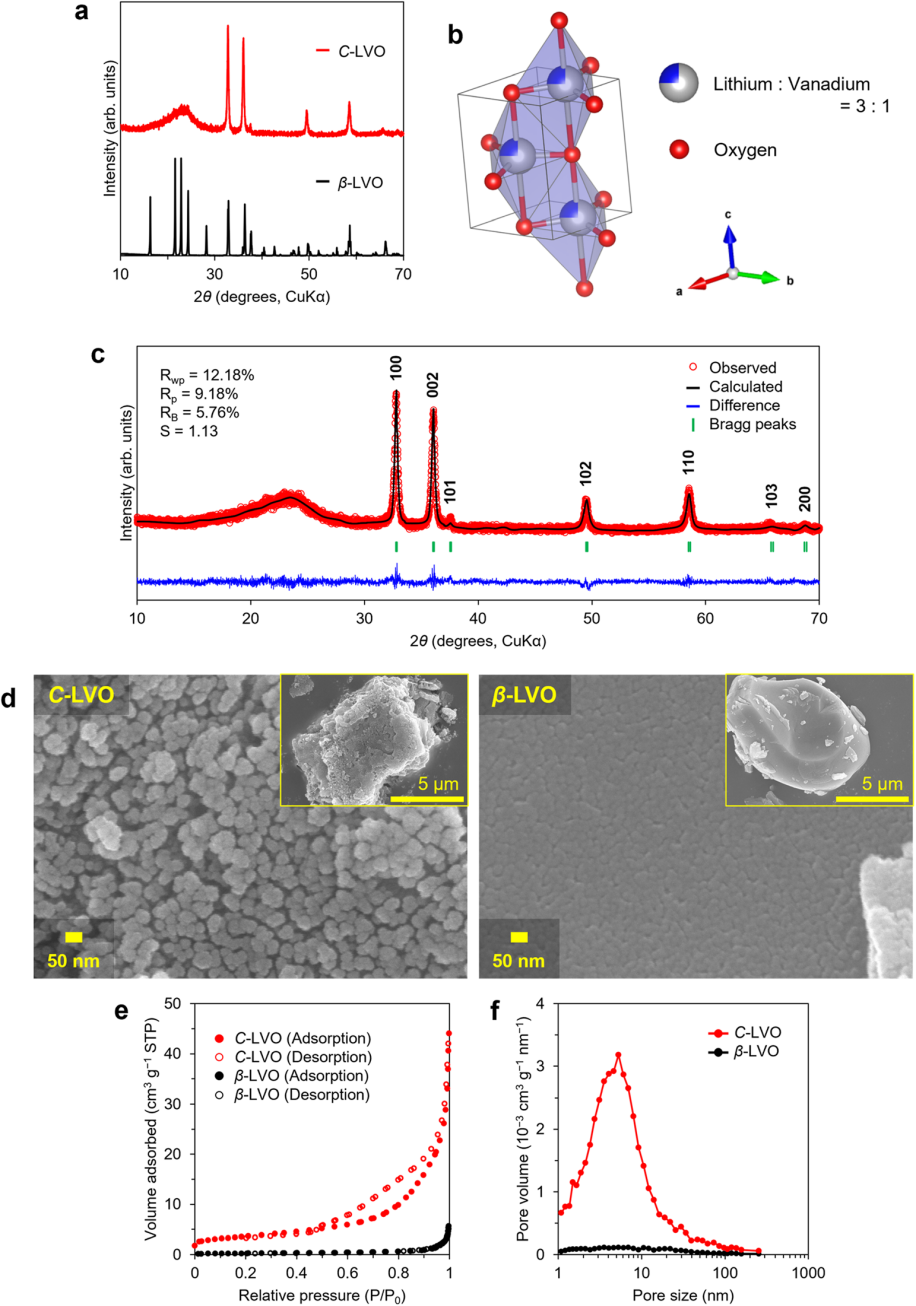
Crystal structure and physical properties. (a) XRD patterns of the powder synthesized *via* solution-based reactions (red line) and solid-state reactions (black line). (b) Rietveld refinement of the powder synthesized through a solution-based reaction. (c) Crystal structure of C-LVO. (d) SEM images of C-LVO (left) and β-LVO (right). The corresponding reduced views are presented in the inset. (e) N_2_ adsorption–desorption isotherms of C-LVO and β-LVO. (f) Porous distribution calculated by the BJH method.

The XRD patterns of LVO crystallized by controlling the drying time in the rotary evaporator to less than one minute (abbreviated C-LVO) and β-phase LVO synthesized through the solid-state method as a reference sample (abbreviated β-LVO) were compared (Fig. 2a). C-LVO exhibits a single broad halo pattern in the low-angle region (2*θ* < 30°), whereas β-LVO exhibits five sharp peaks. In addition to the halo pattern, C-LVO exhibits several sharp peaks at positions similar to those of the β-LVO peaks in the high-angle region (2*θ* > 30°). The characteristic halo pattern is similar to the XRD patterns of cation-disordered LVO,^31,50^ suggesting that it belongs to the same structure class. Therefore, Rietveld refinement was performed of C-LVO by applying a crystal structure model (space group: *P*6_3_*mc*) where the cation sites are shared and occupied by Li and V (Fig. 2b and SI Table 2). As a result, the application of space group *P*6_3_*mc* leads to a high reliability factor, which indicates that C-LVO has a cation-disordered LVO crystal structure. In the crystal structure of C-LVO, oxygen atoms form a hexagonal close-packed structure, with Li and V occupying the centre of the TbP site created by five oxygen atoms in a 3 : 1 ratio (Fig. 2c and S4). In contrast, β-LVO also has a hexagonal close-packed oxygen framework, where Li and V are regularly accommodated in the Td site formed by four oxygen atoms, indicating a difference in each cation accommodation site. In the X-ray photoelectron spectroscopy (XPS), both the C-LVO and β-LVO showed the V 2p_3/2_ peak arising from the vanadium in the pentavalent state (SI Fig. 5). While both the C-LVO and β-LVO particles were µm in size according to the scanning electron microscopy (SEM) observations, the magnified images revealed distinct surface morphologies (Fig. 2d). The C-LVO crystallized with a rotary evaporator exhibited a surface comprising aggregated spherical nanoparticles approximately 50 nm in diameter, whereas the β-LVO prepared *via* high-temperature calcination exhibited a smooth and uniform surface. Detailed surface structure analysis was performed by N_2_ adsorption tests (Fig. 2e). In accordance with the International Union of Pure and Applied Chemistry (IUPAC) classification of N_2_ adsorption isotherms,^57^ C-LVO and β-LVO were categorized as types IV (H3 hysteresis loop) and III, respectively. Adsorption/desorption hysteresis observed in C-LVO above a relative pressure of 0.5 suggests the presence of mesopores. The type III isotherm for β-LVO suggests a nonporous structure, which is consistent with the SEM image. Pore size distributions calculated by the Barrett–Joyner–Halenda (BJH) method revealed that C-LVO has pores smaller than 10 nm (Fig. 2f). According to the Brunauer–Emmett–Teller (BET) method within the relative pressure range of 0.05–0.30, the specific surface areas of C-LVO and β-LVO were 12.63 and 0.89 m^2^ g^−1^, respectively, with C-LVO exhibiting a specific surface area more than 10 times greater than that of β-LVO (SI Fig. 6). The increased surface area due to the formation of nanoparticles and mesopores could increase electrolyte accessibility and facilitate fast Li^+^ insertion/extraction.

### Stability of the crystal structure

**Fig. 3:**
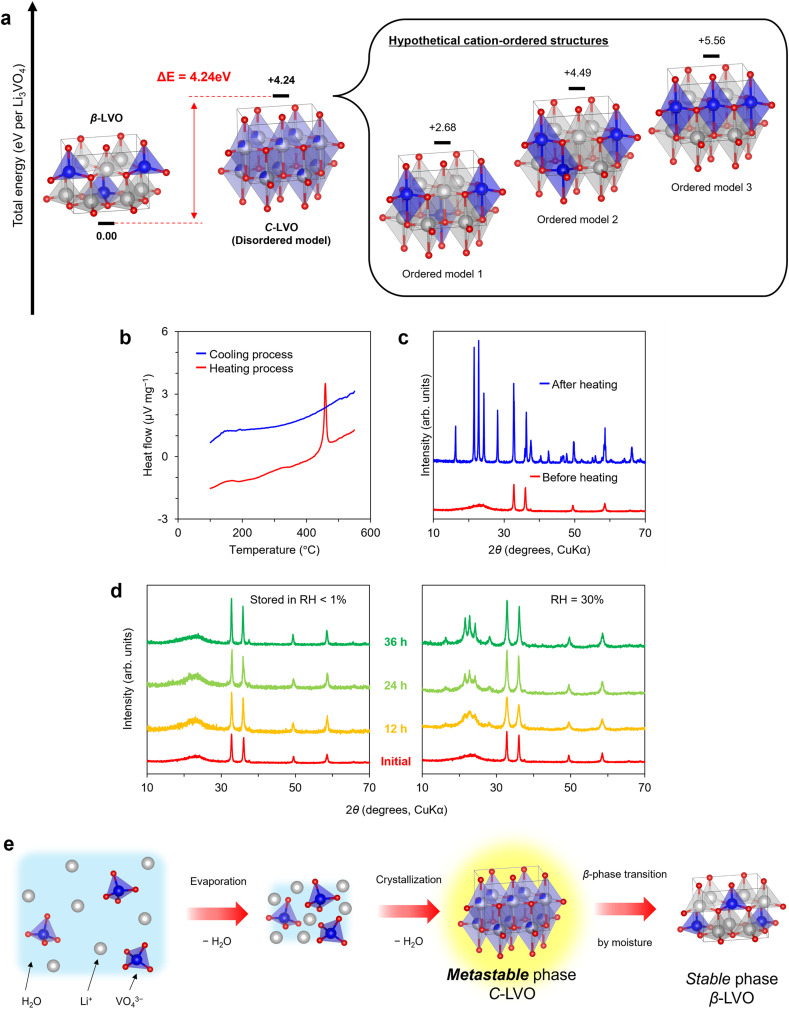
Stability of the crystal structure. (a) Comparison of the total energies of C-LVO and β-LVO. (b) Differential thermal analysis of C-LVO. (c) XRD patterns of the sample before and after differential thermal analysis. (d) XRD patterns of the C-LVO stored under different humidity conditions. (e) Estimation of the mechanism by which C-LVO forms from the aqueous LVO precursor solution.

As the peaks originating from β-LVO emerged in the XRD patterns of C-LVO when C-LVO was stored in air, the structural stabilities of these structures were evaluated computationally and experimentally. It is difficult to represent disordered cation sites because each atom must be assigned an explicit elemental potential in first-principles calculations. Virtual crystal approximation (VCA)^58^ and coherent potential approximation (CPA)^59,60^ enable the representation of several potentials for a single atom. However, these approaches are not suitable for LVO because of the significant differences in electronic states between Li^+^ and V^5+^. Therefore, hypothetical cation-ordered models were adopted for calculation-based evaluation. A Li_6_V_2_O_8_ unit cell (space group: *P*1) was assembled from CIF data to match the number of atoms to compare C-LVO and β-LVO (SI Fig. 7 and Table S3). With respect to the VO_5_ trigonal bipyramid, there are three configurations in C-LVO's Li_6_V_2_O_8_: a model in which VO_5_ units are separated (order model 1), a model in which VO_5_ units share points and edges (order model 2), and a model in which VO_5_ units share points (order model 3). The simulated XRD patterns of the ordered models showed peaks below 30°, whereas such peaks were absent in the disordered model (SI Fig. 8a). These peaks below 30° are greatly influenced by cation sites and disappear when all the cation sites are equivalent. For instance, in models where all the cation sites were set up with either lithium or vanadium, no peaks were detected below 30° in the simulations, and these results resembled those of the disordered model, although the intensity ratios differed (SI Fig. 8b). The disordered model indicated the absence of peaks below 30° because all the cation sites were occupied equivalently by Li (75%) and V (25%). In the actual crystal structure, it is suggested that either Li or V explicitly occupies the cation sites, as in the ordered model at the microscopic scale, and that these structures behave equivalently at the macroscopic scale by being averaged. Hence, we defined the energy of the disordered model as the average of the energy from three ordered models. SCF calculations revealed that the energies of all three ordered models were greater than that of β-LVO, with their average being approximately 4.24 eV greater than that of β-LVO, indicating the instability of C-LVO (Fig. 3a). For experimental evaluation, the thermal stability of C-LVO was evaluated with differential thermal analysis (DTA). C-LVO indicated an exothermic peak at 450 °C without weight loss during the heating process, corresponding to a thermally induced structural phase transition. In the subsequent cooling process, no DTA peak was observed, suggesting an irreversible phase transition (Fig. 3b). Furthermore, the XRD pattern of the sample after the DTA measurement revealed that of β-LVO (Fig. 3c). The exothermic and irreversible structural phase transition suggests that the potential energy of C-LVO is greater than that of β-LVO, which agrees with the calculation results. The results of the calculations and experiments revealed that the cation-disordered structure is a kinetically stabilized metastable phase, whereas the β-phase is a thermodynamically stable phase. Similar behaviour has been reported for metastable cation-disordered Li_2_M(M = Ti, Mn)O_3_, which undergoes an irreversible phase transition to a thermodynamically stable cation-ordered structure upon heat treatment.^61^ Additionally, the influence of moisture on C-LVO was investigated by controlling the relative humidity (RH < 1%, = 30), and XRD patterns were recorded every 12 hours (Fig. 3d). After 12 hours, peaks derived from β-LVO at lower angles (2*θ* < 30°) emerged in the patterns obtained for C-LVO stored at RH = 30%, and their intensities increased over time. In contrast, the C-LVO stored at a RH < 1% always maintained its broad halo pattern in the lower-angle region (2*θ* < 30°). One example of a crystal with similar behaviour is metastable perovskite-type CsPbI_3_; the adsorption of moisture on the crystal surface lowers the activation barrier, facilitating a transition to the thermodynamically stable cubic phase CsPbI_3_.^62,63^ Consequently, it is hypothesized that C-LVO adsorbs atmospheric moisture, which catalytically induces a structural phase transition to the β-phase at room temperature. Further investigation revealed the surface morphologies of C-LVO stored at RH = 30% for 36 hours (abbreviated moisture-treated LVO) through SEM observations and N_2_ adsorption tests (SI Fig. 9). Although slight aggregation was observed, the particles remained in the nanoscale size range, as revealed by SEM observations, and exhibited a specific surface area of 9.18 m^2^ g^−1^ as determined by the BET method based on N_2_ adsorption. The morphologies of moisture-treated LVO are similar to those of C-LVO; however, its crystal structure exhibits the β-phase, suggesting that moisture influences only the crystal structure.

On the basis of these results, a comprehensive mechanism was proposed (Fig. 3e). When V_2_O_5_ is added to a high-pH LiOH aqueous solution, orthovanadate (VO_4_^3−^ oxyanion) forms. At this stage, the aqueous precursor solution contains the essential components of LVO (Li^+^ and VO_4_^3−^). In the drying process involving the evaporator, water is expelled from the system through evaporation, resulting in Li^+^ and VO_4_^3−^ occurring in a minimally solvated state. Further enforced dehydration leads to the precipitation of crystals. This leads to the formation of a metastable cation-disordered structure since vanadium can exist in various coordination states. After the cation-disordered structure forms, promptly removing adsorbed water maintains the structure, resulting in a phase transition to the thermodynamically stable β-phase. Since the rotary evaporator alone does not sufficiently remove moisture at this stage, a rapid dehydration process using vacuum drying becomes a key factor in the synthesis technique. The C-LVO obtained by controlling the drying method is expected to exhibit favourable electrochemical properties because of its crystal structure and surface morphology.

### Electrochemical properties

**Fig. 4:**
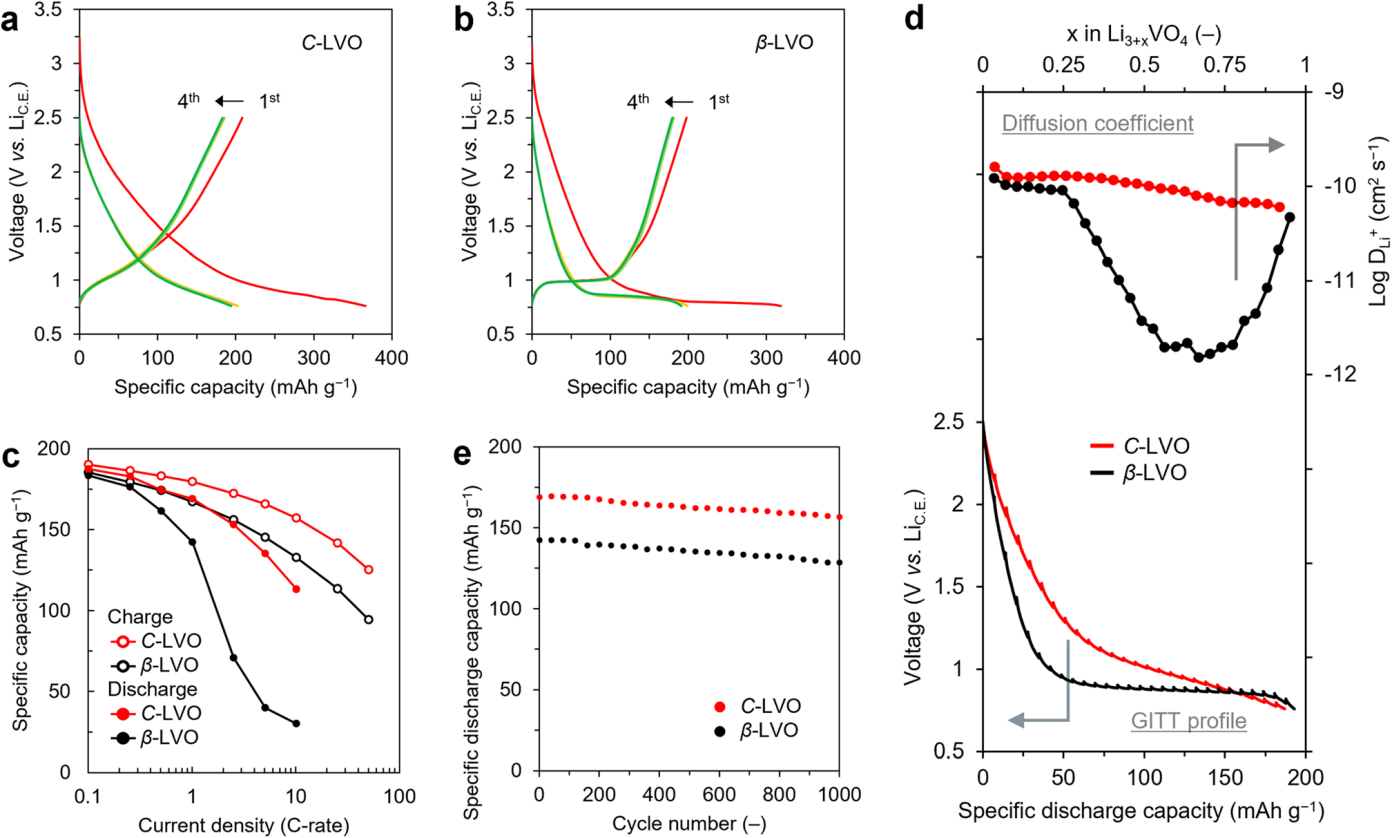
Electrochemical properties discharge/charge curves of (a) C-LVO and (b) β-LVO. (c) Rate capabilities at various current densities. (d) Comparison of diffusion coefficients estimated using GITT. (e) Cyclability test at 1 C-rate.

To evaluate the lithium storage performance of the obtained C-LVO, a half-cell was assembled with lithium metal as the counter electrode, and its performance was compared with that of β-LVO and moisture-treated LVO. The cut-off voltage was set to 0.76 V to prevent changes in properties due to electrochemically induced cation disordering,^29,30^ and the discharge/charge properties were analysed at a current density of 0.1 C-rate (0.02 A g^−1^) (Fig. 4a and b).

In the initial discharge (Li^+^ insertion) process, a high irreversible capacity was observed, which is attributed to side reactions such as electrolyte decomposition and SEI film formation; however, stable curves were obtained during subsequent cycles. During the second cycle, both electrodes exhibited discharge capacities close to 200 mAh g^−1^, which matched the theoretical capacity (197 mAh g^−1^) associated with the single-electron reaction of LVO in the range of 0.76–2.5 V. The discharge/charge curve of β-LVO plateaued at 0.85 and 1.00 V, resembling typical battery-like curves. In comparison, C-LVO displayed capacitor-like sloped discharge/charge curves, revealing characteristic behaviour in cation-disordered LVO.^29–31,50^ d*Q*/d*V* analysis revealed broadened peaks for C-LVO, providing clear evidence of the slope behaviour (SI Fig. 10). The slope observed in the discharge/charge curve is believed to result from cation disordering in the structure.^14,15^ Furthermore, moisture-treated LVO displayed battery-like curves as well as β-LVO, indicating that the crystal structure affects the shape of the discharge/charge curves (SI Fig. 11). The following tests were conducted *via* stabilized cells (precycled cells) after the initial cycle. In our previous reports, we demonstrated that the discharge rate capability of LVO is lower than its charge rate capability.^49^ Therefore, to examine the rate performance in detail, the counter current density was fixed at 0.1 C-rate, and the discharge (Li^+^ insertion) and charge (Li^+^ extraction) processes were assessed separately by varying the current density (Fig. 4c and S12). In the discharge process, β-LVO maintained a high capacity retention at 1 C-rate; however, its capacity retention decreased significantly at higher current densities. The reason for this finding is that β-LVO exhibits a plateau at 0.85 V at 0.1 C-rate, and the discharge curve changes significantly with increasing current density and becomes more strongly affected by the cut-off voltage (0.76 V). Consequently, β-LVO did not demonstrate sufficient capacity within the voltage range of 0.76–2.5 V during the discharge process. The sloped curve for C-LVO was less affected by the cut-off voltage, and minimal changes in the discharge curve were observed, resulting in greater capacity retention. C-LVO exhibited a greater capacity retention than β-LVO did, with the capacity retentions of C-LVO and β-LVO being 60% and 16% at 10 C-rate (2 A g^−1^), respectively. Moisture-treated LVO exhibited a capacity approximately 10 mAh g^−1^ higher than that of β-LVO; however, similar to β-LVO, its capacity retention decreased significantly when the current density exceeded 1 C-rate (SI Fig. 13). In the charging process, these LVO materials showed greater capacity retention in the charging process than in the discharge process, with C-LVO and β-LVO retaining 66% and 51% of their capacities, respectively, at 50 C-rate (10 A g^−1^). Moisture-treated LVO also exhibited a high capacity retention, showing a charge rate capability comparable to that of C-LVO, with a retention of 64% at 50 C-rate. To reveal the improvement of rate capability, the apparent Li^+^ diffusion coefficient (*D*_Li^+^_) was estimated using the galvanostatic intermittent titration technique (GITT) (Fig. 4d and S14). In the discharge process, the *D*_Li^+^_ of β-LVO and moisture-treated LVO began to decline when *x* exceeded 0.3 in Li_3+*x*_VO_4_, while those of C-LVO remained relatively high throughout. This behaviour is consistent with the cation-disordered LVO induced by electrochemical activation.^30^ The *D*_Li^+^_ of C-LVO were enhanced up to 50-fold compared with those of β-LVO, indicating that cation disordering intrinsically contributes to the improvement of *D*_Li^+^_. In both processes, compared with β-LVO, C-LVO significantly improved the rate performance. This improvement is attributed not only to changes in curve shape but also to enhanced diffusion coefficients from the crystal structure, reduced Li^+^ diffusion length within particles due to size reduction, and increased electrolyte accessibility resulting from increased specific surface area. Cyclability of the precycled cells was assessed at a current density of 1 C-rate (0.2 A g^−1^) (Fig. 4e and S15). Each electrode has the same capacity, which is the value obtained at 1 C-rate in the charging rate test. This is attributed to the dependence of capacity on the charging process, which is the rate-limiting step at a constant current. Unlike β-LVO, C-LVO exhibited a capacity exceeding 30 mAh g^−1^. Both electrodes demonstrated stable cycling because of their high coulombic efficiency, and the capacities of C-LVO and β-LVO were 92.7% and 90.3%, respectively, after 1000 cycles. A comparison of the shapes of the discharge/charge curves from the 1st and 1000th cycles revealed minimal changes, demonstrating the high electrochemical stability of C-LVO.

## Conclusions

In this study, by clarifying the technical factors related to drying conditions, we demonstrate low-temperature one-pot green synthesis of C-LVO with high electrochemical performance *via* a reaction with an eco-friendly solution using a rotary evaporator. The chemical reaction equation for this reaction is as follows (eqn (1)):

1 6LiOH–H_2_O + V_2_O_5_ → 2Li_3_VO_4_ + 9H_2_O

**Fig. 5:**
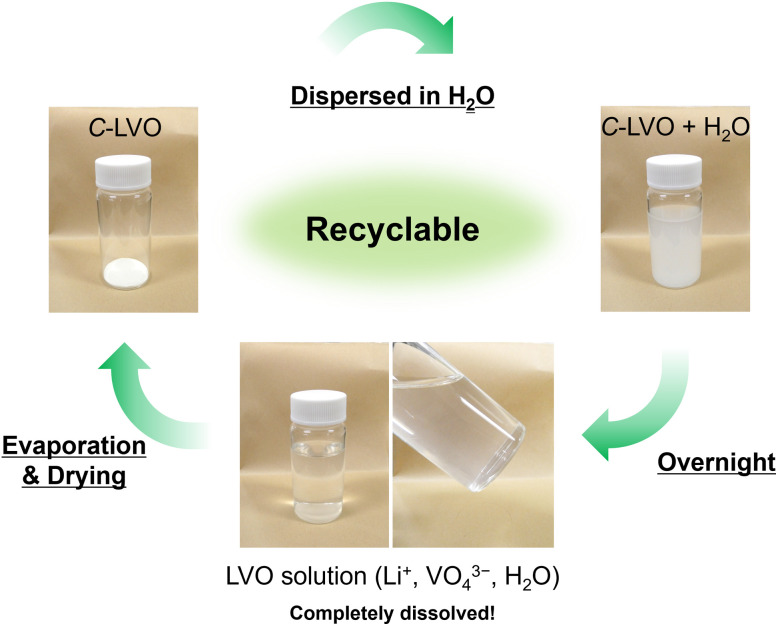
Dissolution of C-LVO in H_2_O and recycled C-LVO.

This reaction is an eco-friendly reaction that achieves many of the 12 principles of green chemistry.^64^ It involves the use of water as a solvent and consists of a simple synthesis process of dissolution and drying at 40 °C, resulting in high atom efficiency without the use of auxiliaries. Water as a byproduct is recovered during drying, so we can obtain only C-LVO. Interestingly, LVO is soluble in water, and dissolving it returns it to Li^+^ and VO_4_^3−^. This suggests that easy resource recovery is feasible, making it a promising recyclable next-generation anode material (Fig. 5).

In synthesis methods such as hydrothermal reactions, crystals grow slowly in the long term while selecting thermodynamically stable orientations and thus yielding thermodynamically stable phases. In contrast, crystallization *via* evaporation using drying equipment forcibly separates solutes from the solution in a short time, which is believed to lead to kinetically stabilized metastable phases. Indeed, there have been reports showing that evaporation-driven crystallization induces metastable phases.^65^ Given that vanadium is a transition metal with various coordination structures, it is suggested that the evaporation process promotes the formation of cation-disordered LVO as a kinetically stabilized structure. Although cation-disordered LVO is readily transformed into thermodynamically stable phases because of water adsorbed on its surface, the spray-drying technique,^50^ which enables instantaneous crystallization and immediate separation from moisture, is suitable for synthesizing a kinetically stabilized structure. Moreover, spray dryers are scalable equipment widely used in industry, making large-scale commercialization highly promising. However, synthesis could also be achieved by combining drying with a rotary evaporator and subsequent vacuum drying for rapid moisture removal.

The synthesis method involving the preparation of components in aqueous solution followed by recovery through drying is rational and holds promise for application to a wide range of materials, not limited to LVO, since many transition metal species can form oxoanions in aqueous solutions at high pH.^56^ In addition, this methodology provides a broader community of researchers with the opportunity to discover high-performance next-generation materials with an eco-friendly method since kinetically stable metastable structures can be synthesised using a standard laboratory apparatus.

## Author contributions

T. Kondo conceived the research concept, prepared the materials, measured the physical and electrochemical properties, conducted the theoretical calculations, and wrote the manuscript. S. Kawaguchi conducted the theoretical calculations and participated in the discussion. N. Takeshima participated in the discussion and corrected the manuscript. S. Igari measured the electrochemical properties and XPS spectra, and corrected the manuscript. S. Tatsumi measured the electrochemical properties. K. Akiyama performed Raman spectroscopy. K. Machida corrected the manuscript and administered the projects. S. Takeda administered the projects. All the authors contributed comments on this work.

## Conflicts of interest

There are no conflicts of interest to declare.

## Supplementary Material

RA-016-D6RA01593J-s001

## Data Availability

All data supporting the findings of this study are available within this paper and its supplementary information (SI). Additional data related to this paper are available upon reasonable request from the corresponding author, within the limits permitted by our company regulations. Supplementary information: additional figures, tables, and detailed experimental and computational procedures supporting the results discussed in the main text. See DOI: https://doi.org/10.1039/d6ra01593j.
