# Supplementary material for: Green one-pot synthesis of recyclable cation-disordered Li3VO4 below 40 °C for high-rate anode materials
Source: RSC Adv. 2026 Mar 18;16(16):14975–83. doi: 10.1039/d6ra01593j (PMC12998446; doi:10.1039/d6ra01593j)
Supplement: RA-016-D6RA01593J-s001 [file RA-016-D6RA01593J-s001.pdf]

Supplementary Information (SI) for RSC Advances.
This journal is © The Royal Society of Chemistry 2026

Supplementary Information

Green one-pot synthesis of recyclable cation-disordered Li_3VO_4 below 40 °C for high-rate anode materials

Tatsuya Kondo[1,2,*], Sota Kawaguchi[1], Naoto Takeshima[2], Shuto Igari[1], Satoyuki Tatsumi[2], Kaori Akiyama[2], Kenji Machida[2], Sekihiro Takeda[2]

[1]Basic Research Center, Nippon Chemi-Con Corporation, KSP R&D C1025, 3-2-1 Sakado, Takatsu-ku, Kawasaki-shi, Kanagawa 213-0012, Japan

[2]Basic Research Center, Nippon Chemi-Con Corporation, Takahagi, Ibaraki 318-8505, Japan

***Corresponding author:** E-mail: t-kondo@nippon.chemi-con.co.jp

Methods

Synthesis of Li_3VO_4

LiOH-H_2O (30 mmol, FUJIFILM Wako Pure Chemical Corporation) was dissolved completely in 100 mL of distilled water. Subsequently, V_2O_5 (5 mmol, Kanto Chemical Co., Inc.) was slowly added in 10 portions, ensuring complete dissolution to prepare a colourless and transparent 100 mM LVO aqueous precursor solution (pH = 11.8). Excess Li^+ may preferentially lead to the formation of different compounds. The aqueous precursor solution was dried (evaporation rate: > 1.7 mL min^{-1}) under reduced pressure (< 1000 Pa) at 40 °C using a rotary evaporator (N-1110, TOKYO RIKAKIKAI CO., Ltd. (EYELA)) and a dry diaphragm vacuum pump (DTC-41, ULVAC KIKO, Inc.). Crystallization occurred simultaneously with the complete evaporation of water; accordingly, the evaporator was rapidly purged with dry nitrogen. The generated crystals were transferred to a vacuum dryer within one minute and dried at 40 °C under vacuum (< 0.67 Pa) for 1 hour using an oil rotary vacuum pump (GLD-137CC, ULVAC KIKO, Inc.), resulting in the synthesis of *C*-LVO. To maintain the cation-disordered structure, it was stored in a glove box filled with argon gas until use. For comparison, *β*-LVO was prepared as a reference sample synthesized by the conventional solid-state method.[1]

Physical characterization

Raman spectra were measured with a Raman spectrometer (NRS-5500, Jasco Corporation) by placing a droplet of aqueous LVO precursor solution on a copper foil and scanning in the range of 200 to 4000 cm^{-1} with a 532 nm laser. Powder XRD patterns were obtained using an X-ray diffractometer (Ultima IV, Rigaku Corporation) with CuKα radiation (λ = 0.15418 nm) in the 2θ range of 10–70°. The scan speed was set to 2° min^{-1}, and the resolution was 0.02° $step^{-1}$ in continuous mode. To clarify the detailed crystal structure, Rietveld refinement was performed using RIETAN-FP[2] for the obtained XRD patterns after the baseline was corrected using WinPLOTR.[3] The CIF files of the space groups $P6_3mc$ and $Pmn2_1$ were applied to *C*-LVO and *β*-LVO, respectively. The crystal structures were depicted using VESTA software.[4] XPS spectra were collected on an X-ray photoelectron spectrometer (ESCA-3400, Shimadzu Corporation) with an emission current of 15 mA and an accelerating high tension of 10 kV, using a Mg anode (step width: 0.01 eV, dwell time: 259.7 ms). The sample was prepared by adhering it onto a carbon tape, and the C 1s peak of adventitious carbon at 284.8 eV was used as the reference. SEM images were recorded using an FE-SEM system (S-4700, Hitachi High-Tech Corporation) with an accelerating voltage of 10 kV. N_2

adsorption–desorption isotherms were measured at 77 K using a high-precision gas/vapour adsorption analyser (BELSORP-max, BEL Japan, Inc.). The amount of adsorbed water was determined by heating to 200 °C at a rate of 5 °C min^{-1} using a TG/DTA simultaneous measuring instrument (DTG-60A, Shimadzu Corporation). To evaluate its thermal stability, the *C*-LVO sample was heated from 100 to 550 °C at 5 °C min^{-1} and then cooled to 100 °C at the same rate, after which DTA was performed.

Theoretical calculations

To compare *C*-LVO and *β*-LVO, a CIF file of the $Li_6V_2O_8$ model (space group: *P*1) was established with the same number of atoms. First-principles calculations were performed via the Quantum ESPRESSO package (Ver. 6. 7 Max)[5,6] with density functional theory (DFT). Each atom (Li, V, O) was assigned the projector augmented wave (PAW) pseudopotential with the Perdew–Burke–Ernzerhof (PBE) generalized gradient approximation (GGA)-stored Quantum ESPRESSO pseudopotential database library. The energy cut-off was set to 51 Ry to expand the wave functions and 642 Ry for the charge density with a 10^{-8} Ry energy convergence threshold for the SCF. The Brillouin zone was sampled at 3 × 3 × 3 k-points with 0.01 Ry of Methfessel–Paxton smearing for the unit cell. The stability of

each structure was evaluated by comparison to the total energy obtained from the SCF calculations.

Electrochemical characterization

For electrochemical evaluation, a laminate-type half-cell was assembled in a glovebox filled with argon gas. The slurry of the working electrode was prepared by mixing 70 wt% active material, 20 wt% multiwalled carbon nanotubes (AMC; Ube Industries, Ltd.) as the conductive agent and polyvinylidene fluoride (KF Polymer L #9305; Kureha Corporation) as the binder in NMP (*N*-methyl-2-pyrrolidone). The working electrode (weight: 1.0 mg cm^{-2} per active material; thickness: 7 μm) was prepared by coating the resulting slurry onto Cu foil and drying it at a temperature of 60 °C under vacuum. The laminated cell was assembled with a working electrode, 100 μm of Li metal foil (Honjo Metal Co., Ltd.) as the counter electrode and a 15 μm-thick polypropylene film (Toray Industries, Inc.) as the separator. Prior to lamination, the electrolyte solution (LIPASTE; Tomiyama Pure Chemical Industries, Ltd.) comprising 1.0 mol L^{-1} $LiPF_6$ in ethylene carbonate (EC) and diethyl carbonate (DEC) (1:1 in volume) was poured into the laminated cell. Charge/discharge properties were measured on a battery charge/discharge system (HJ1005SD8, Hokuto Denko CORPORATION) at 27 °C. Measurements were performed in

constant current mode in the voltage range of 0.76–2.5 V *vs.* $Li_{C.E.}$ to prevent the electrochemical activation of LVO[7]. After 4 charge/discharge cycles at 0.2 A g^{-1} (1 C-rate), rate and cycling tests were performed, and D_{Li^+} was evaluated using GITT proposed by Weppner and Huggins.[8] The acquisition of GITT profiles and the calculation of D_{Li^+} were based on a previously reported method.[1] The rate test was performed at various current densities (0.02, 0.05, 0.1, 0.2, 0.5, 1, 2, 5, and 10 A g^{-1}), while the opposite side of the current density was fixed at 0.02 A g^{-1}. The cycling test was evaluated at 0.2 A g^{-1} (1 C-rate), with the current density of the opposite side also set to the same value.

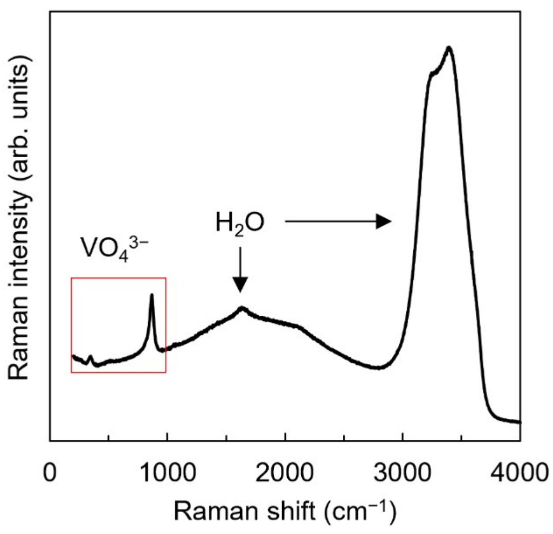

Supplementary Fig. 1. Raman spectra of aqueous LVO precursor solutions within the range of 200 to 4000 cm^{-1}.

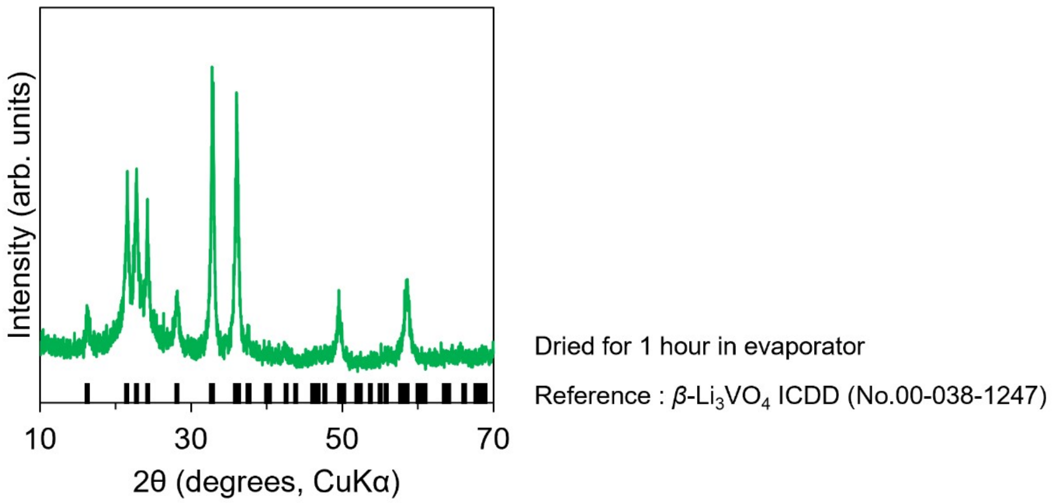

Supplementary Fig. 2. XRD patterns of the powder obtained by drying the aqueous LVO precursor solution.

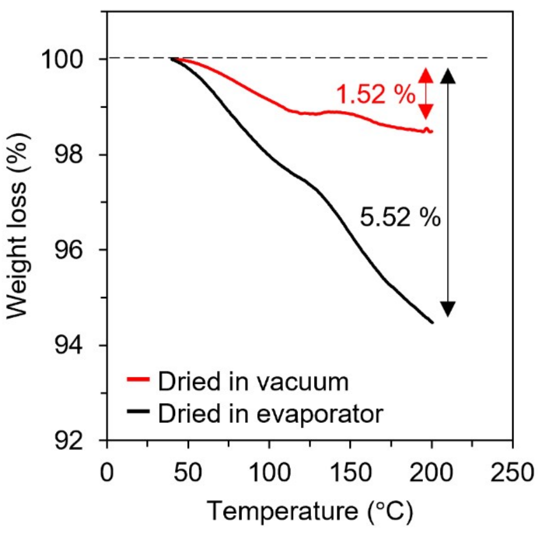

Supplementary Fig. 3. Differences in the amount of adsorbed water for different drying methods.

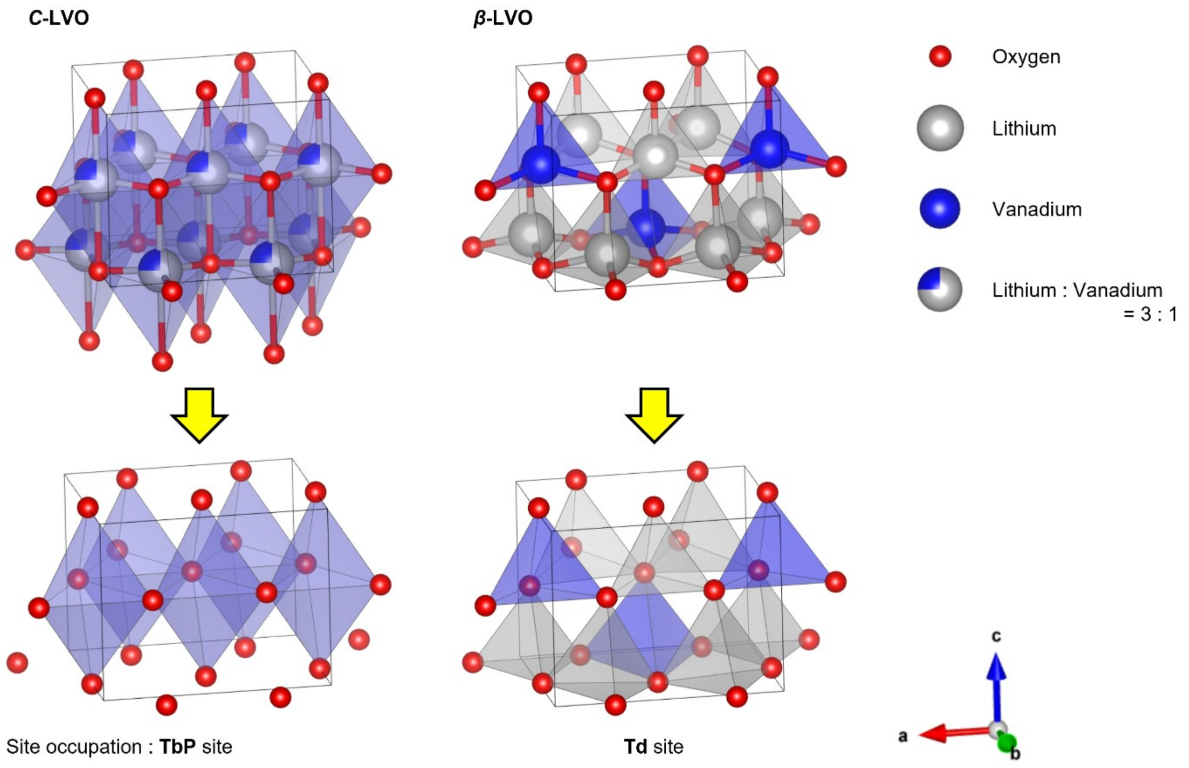

Supplementary Fig. 4. Comparison of the crystal structures of *C*-LVO and *β*-LVO.

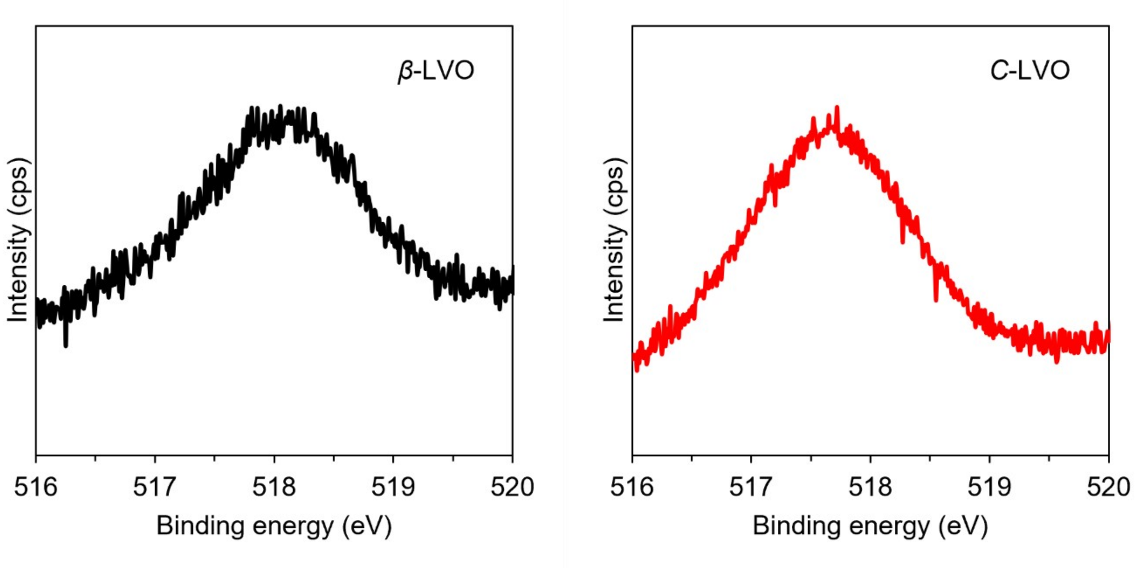

Supplementary Fig. 5. XPS spectra for V $2p_{3/2}$ of *β*-LVO and *C*-LVO.

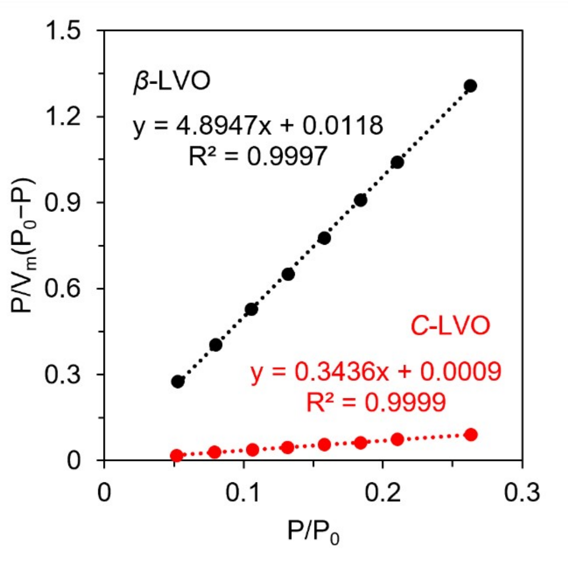

Supplementary Fig. 6. Linear fitted BET plots of *C*-LVO and *β*-LVO within the relative pressure range of 0.05–0.30.

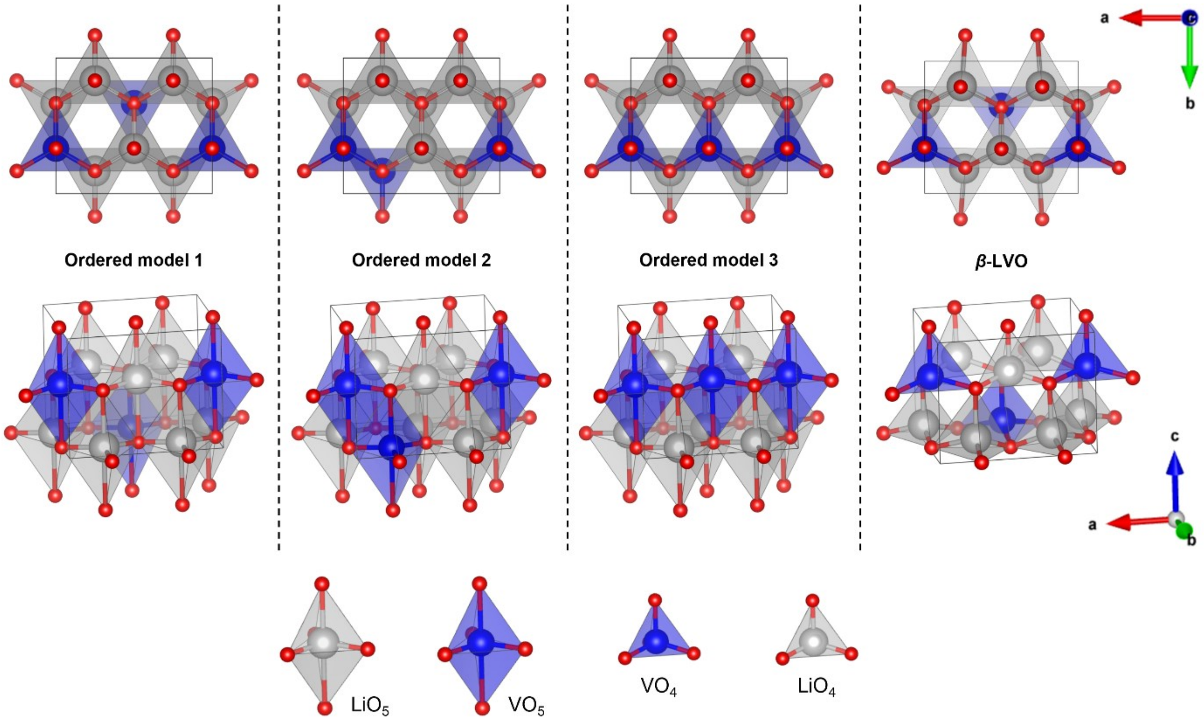

Supplementary Fig. 7. Hypothetical cation-ordered LVO and *β*-LVO models.

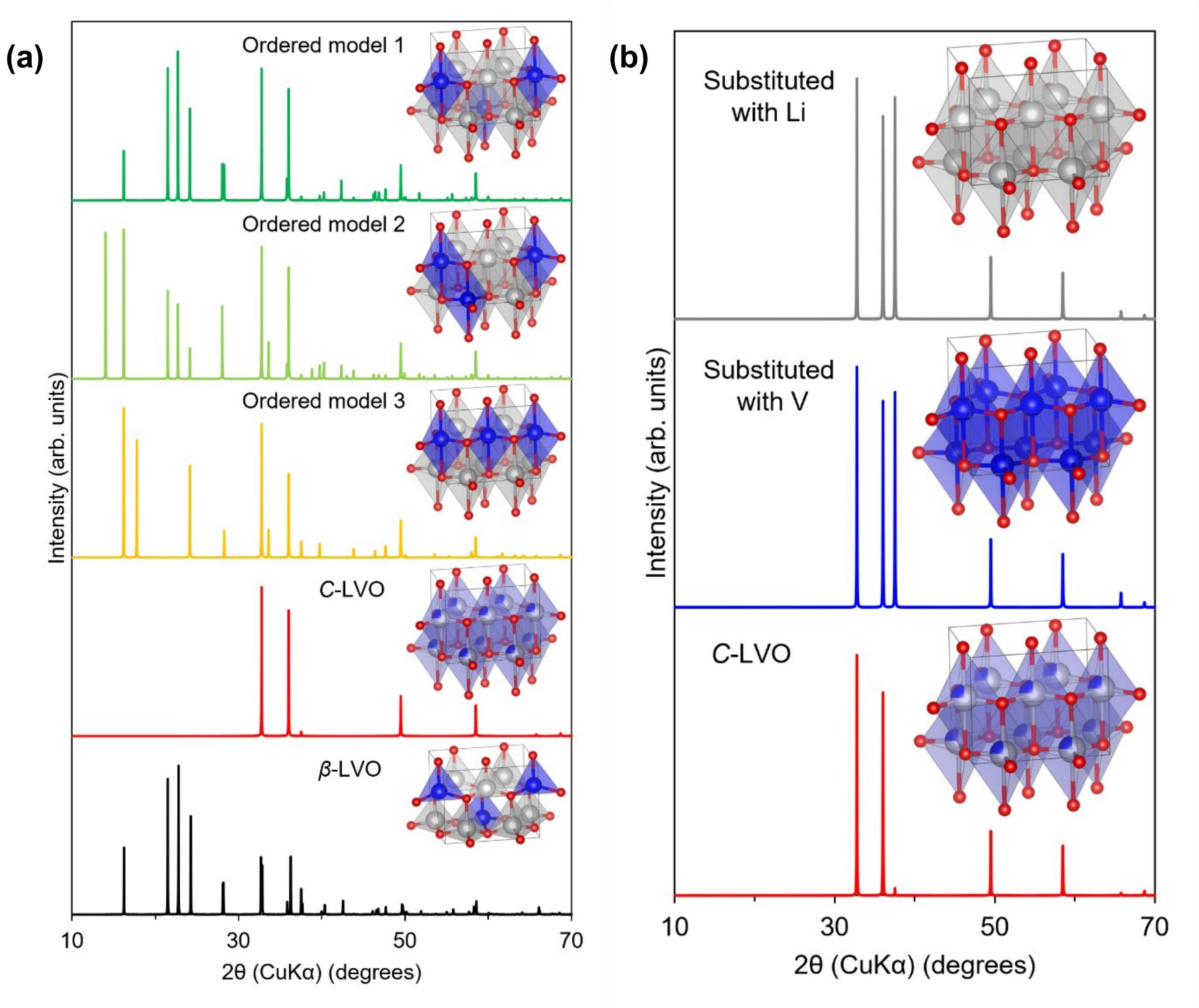

Supplementary Fig. 8. (**a**) Simulated XRD patterns of the ordered model, *C*-LVO, and *β*-LVO. (**b**) Simulated XRD patterns of all the cation site-substituted model.

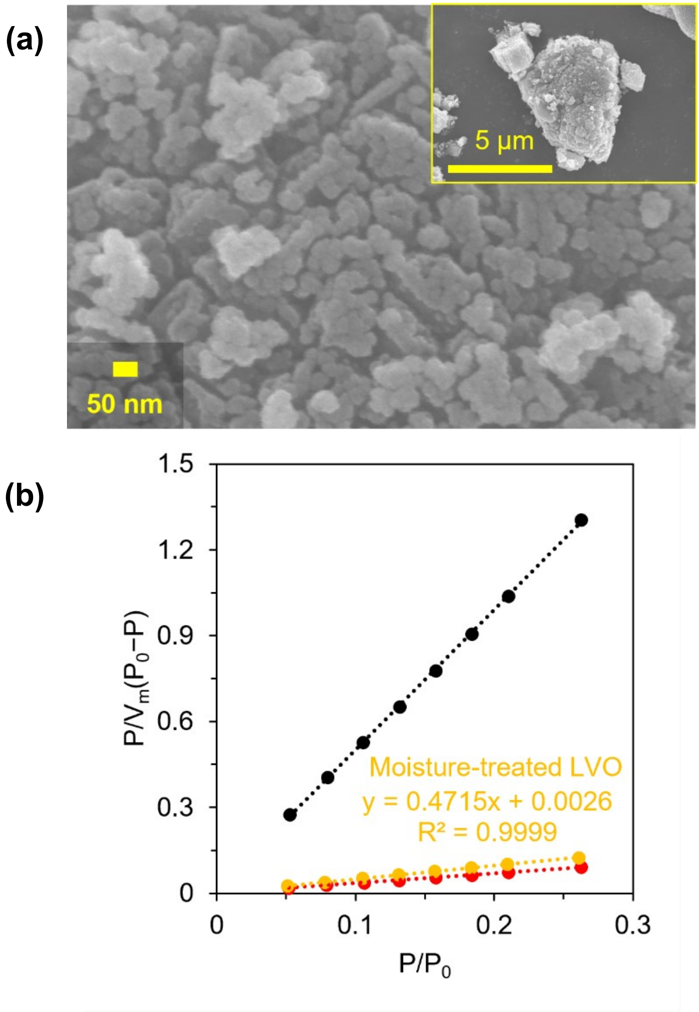

Supplementary Fig. 9. (**a**) SEM images of moisture-treated LVO. (**b**) Linear fitted BET plots of moisture-treated LVO, together with those of *C*-LVO and *β*-LVO.

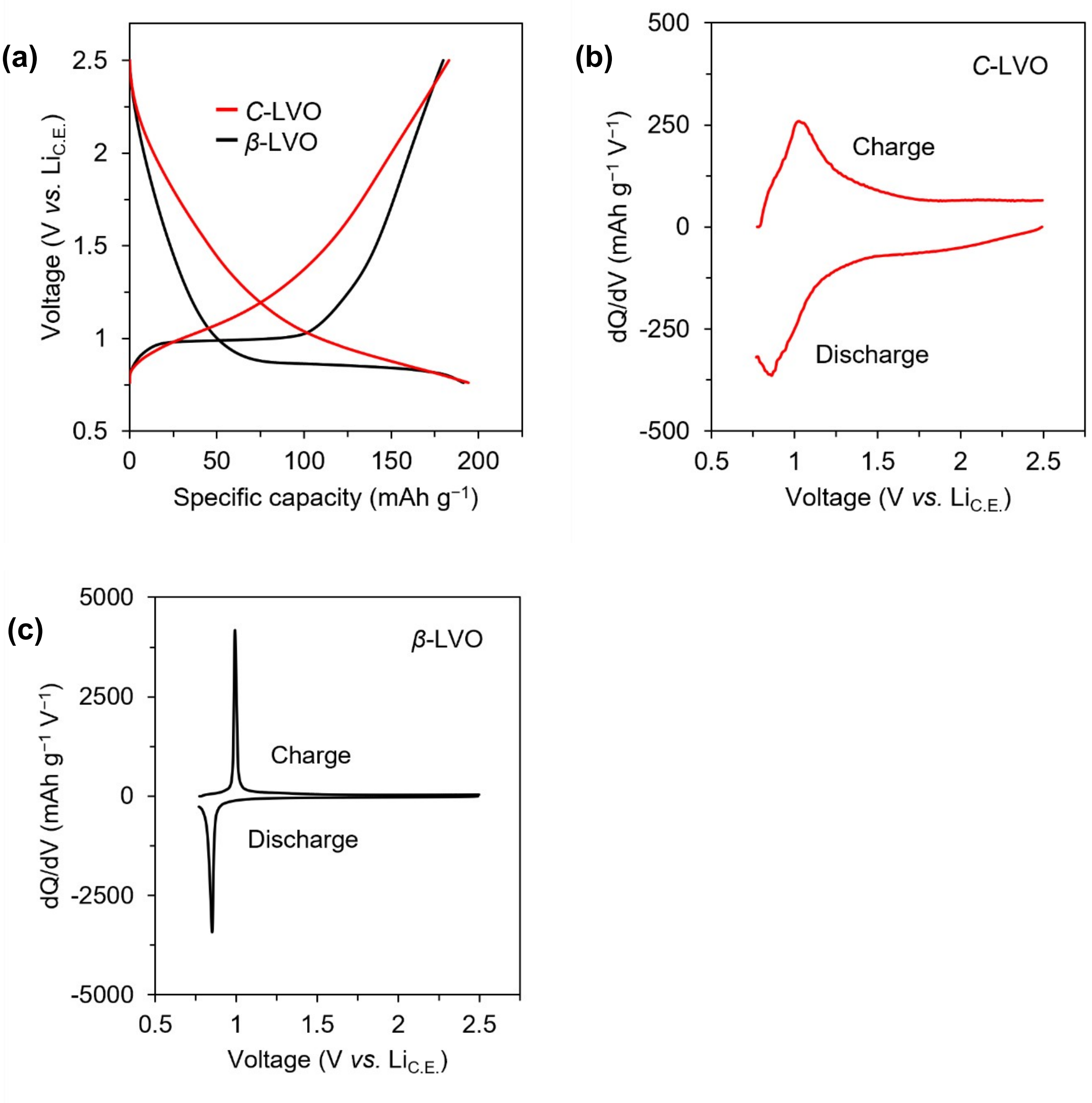

Supplementary Fig. 10. (**a**) Discharge/charge curves of *C*-LVO and *β*-LVO at 0.1 C-rate. dQ/dV curves of (**b**) *C*-LVO and (**c**) *β*-LVO calculated from discharge/charge curves.

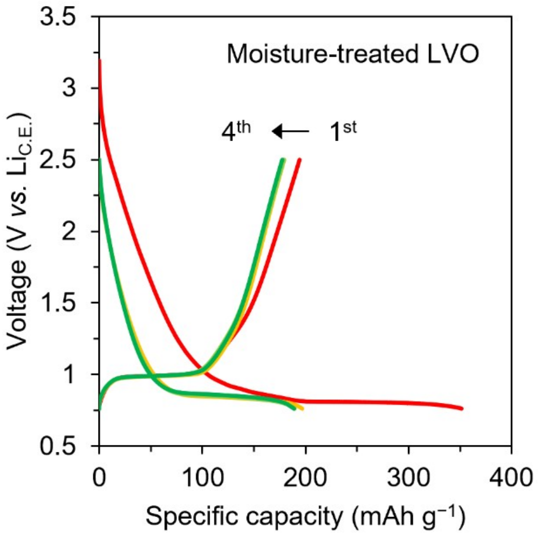

Supplementary Fig. 11. Discharge/charge curves of moisture-treated LVO at 0.1 C-rate.

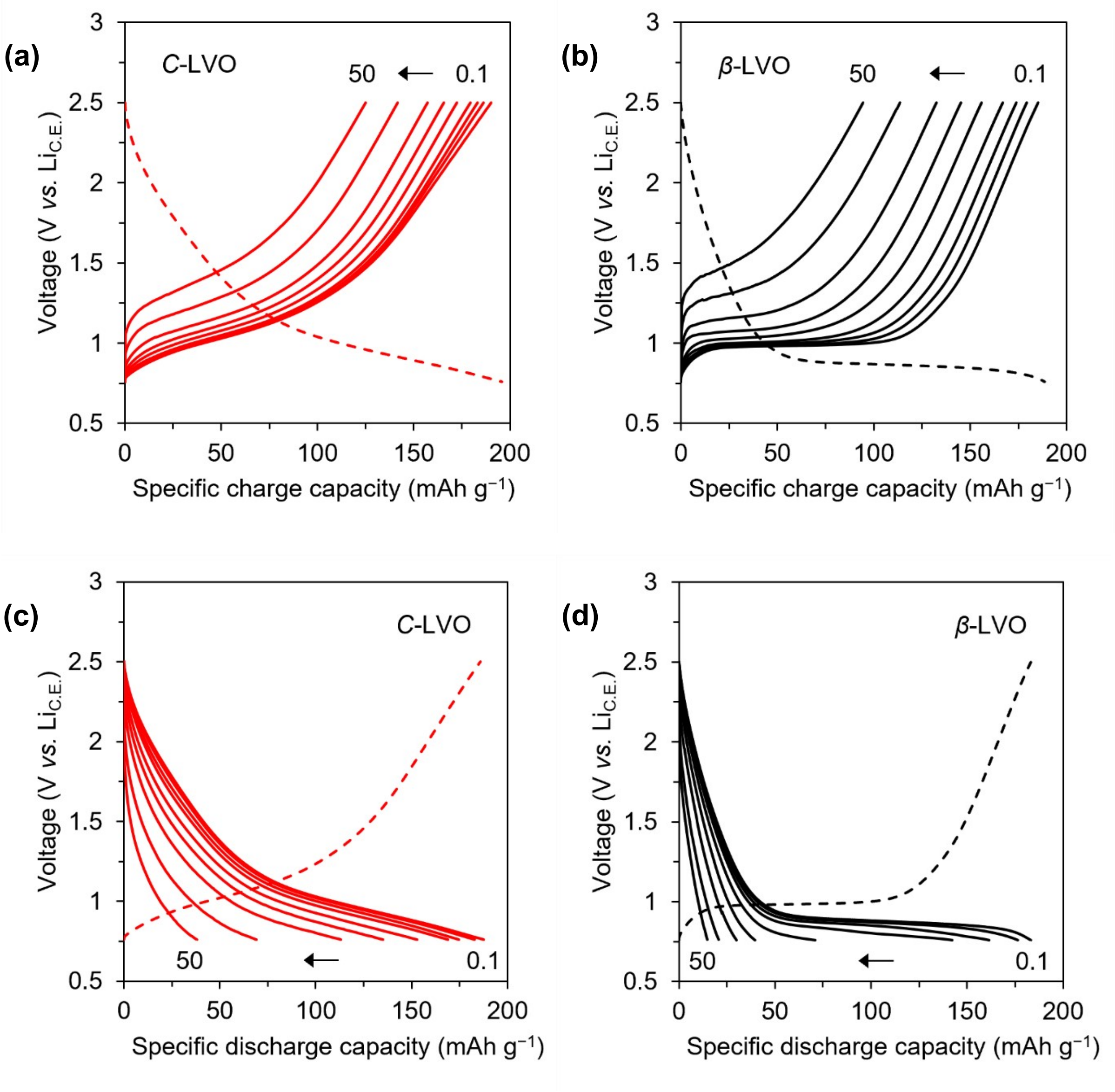

Supplementary Fig. 12. Charge rate capability of (**a**) *C*-LVO and (**b**) *β*-LVO.

Discharge rate capability of (**c**) *C*-LVO and (**d**) *β*-LVO.

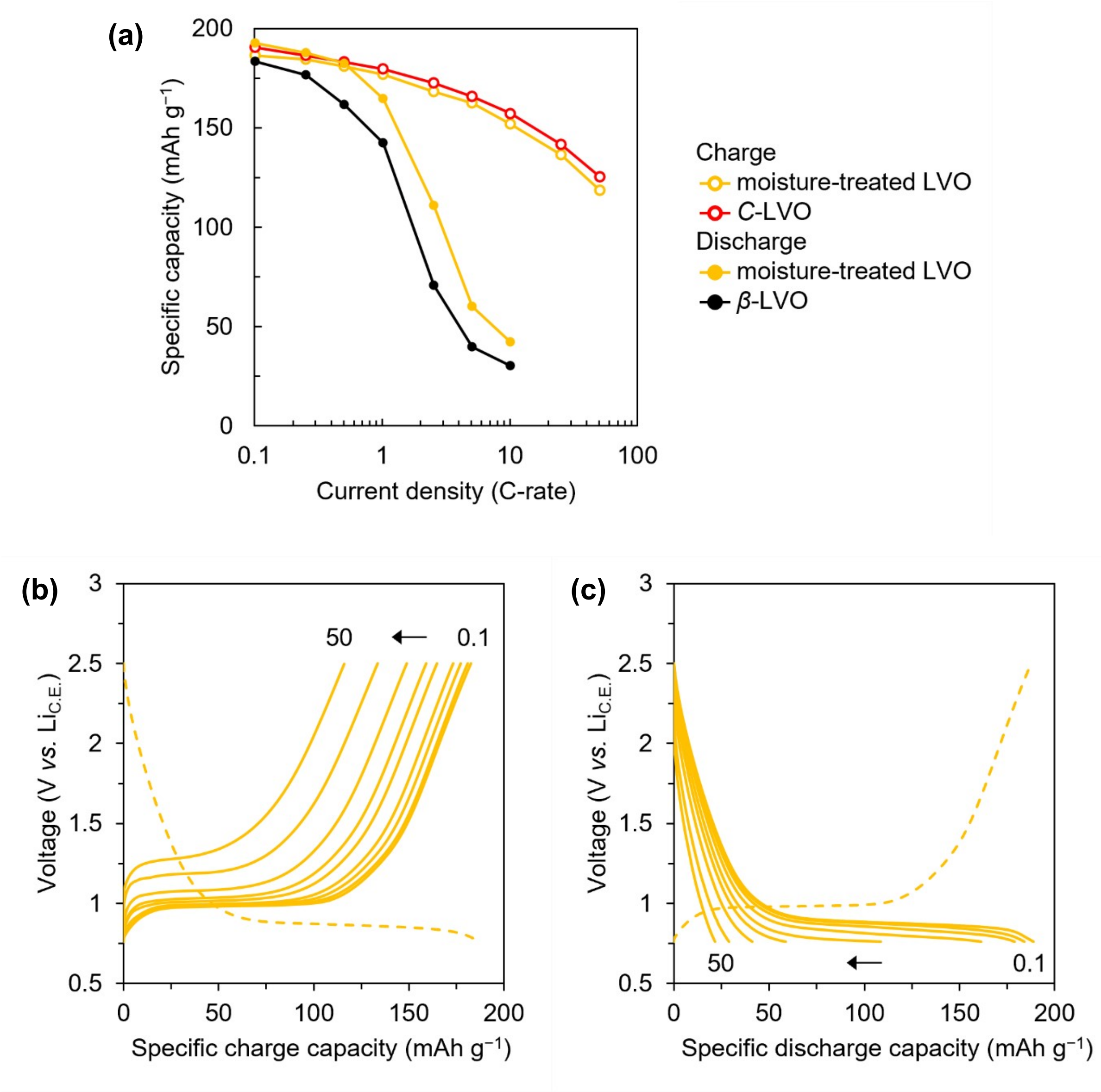

Supplementary Fig. 13. (**a**) Rate capability of moisture-treated LVO. (b) Charge curves and (c) Discharge curves for moisture-treated LVO at various current densities.

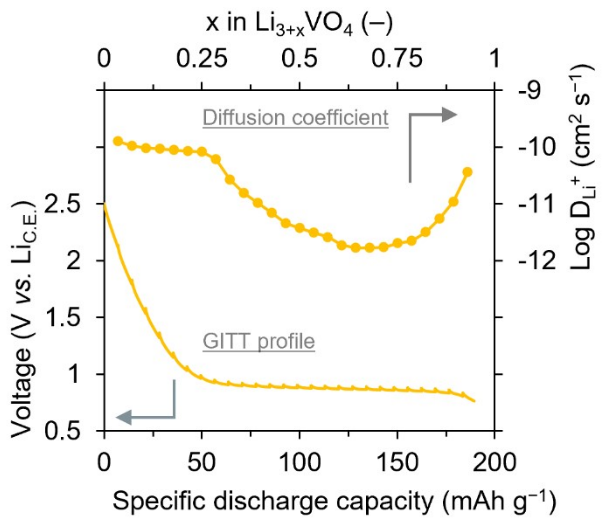

Supplementary Fig. 14. Diffusion coefficients and GITT profile for moisture-treated LVO.

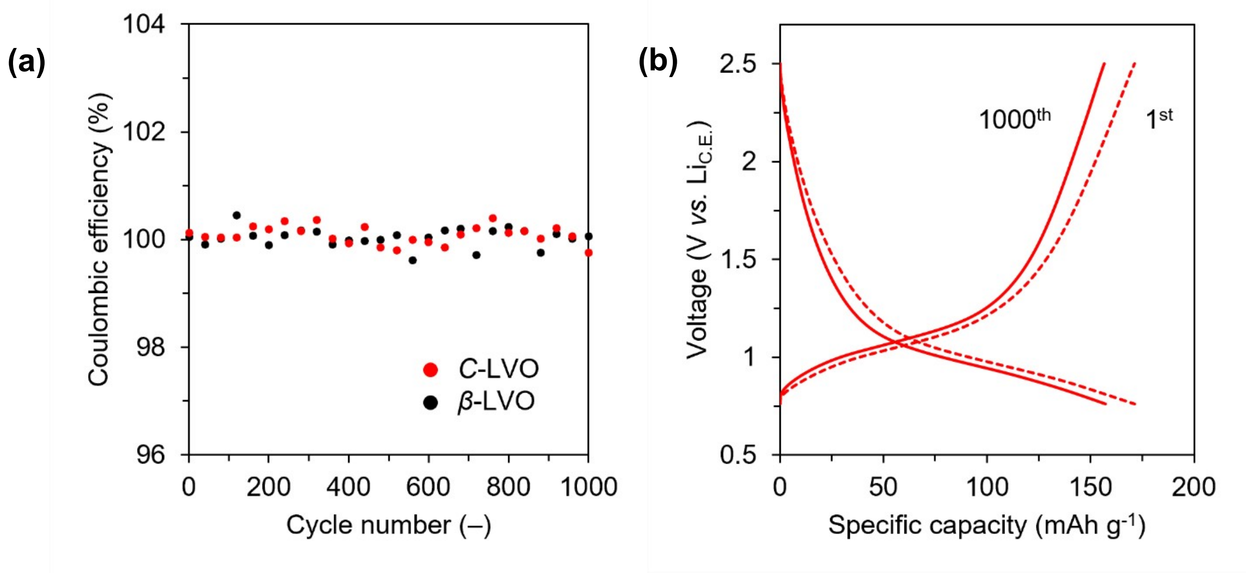

Supplementary Fig. 15. (**a**) Coulombic efficiencies of *C*-LVO and *β*-LVO at 1 C-rate.

(**b**) Comparison of the discharge/charge curves for the 1st and 1000th cycles.

Supplementary Table 1. Comparison of preparation times for cation-disordered LVO.

Methodology[Reference]	Preparation of β-LVO	Cation-disordering
Electrochemical[30]	Required (ca. 15 hours)	160 hours<br>(20 cycles at 0.25 C-rate between 2.5–0.1 V)
Mechanochemical[31]	Required (ca. 13 hours)	36 hours<br>(ball-milling with 600 rpm)
**This work**	**Not required**	**~ 2 hours**<br>**(mixing and drying)**

Supplementary Table 2. Atomic position of *C*-LVO refined via Rietveld analysis of the XRD patterns.

C-LVO (Space group : $P6_3mc$ (186))

Atom	Wyckoff position	*x*	*y*	*z*	Occupancy
Li	2b	0.33333	0.66667	0	0.75
V	2b	0.33333	0.66667	0	0.25
O	2b	0.33333	0.66667	0.47340	1

$a = b = 3.153(3)$ Å, $c = 4.982(3)$ Å, $\alpha = \beta = 90°$, $\gamma = 120°$

Supplementary Table 3. Atomic positions of *C*-LVO and *β*-LVO for the first-principles calculations.

C-Li_3VO_4 (Ordered model 1) (Space group : *P*1 (1))

Atom	Wyckoff position	*x*	*y*	*z*	Occupancy
Li	1a	0.5	0.333333	0.0266	1
Li	1a	0.25	0.833333	0.0266	1
Li	1a	0.75	0.833333	0.0266	1
Li	1a	0.25	0.166667	0.5266	1
Li	1a	0	0.666666	0.5266	1
Li	1a	0.75	0.166667	0.5266	1
V	1a	0	0.333333	0.0266	1
V	1a	0.5	0.666666	0.5266	1
O	1a	0	0.333333	0.5	1
O	1a	0.5	0.333333	0.5	1
O	1a	0.25	0.833333	0.5	1
O	1a	0.75	0.833333	0.5	1
O	1a	0.25	0.166667	0	1
O	1a	0	0.666666	0	1
O	1a	0.75	0.166667	0	1
O	1a	0.5	0.666666	0	1

a = 6.306 Å, b = 5.46116 Å, c = 4.982 Å, $\alpha = \beta = \gamma$ = 90°

β-Li_3VO_4 (Space group : *P*1 (1))

Atom	Wyckoff position	*x*	*y*	*z*	Occupancy
Li	1a	0.5	0.33611	0.1282	1
Li	1a	0.25816	0.81215	0.13612	1
Li	1a	0.74184	0.81215	0.13612	1
Li	1a	0.24184	0.18785	0.63612	1
Li	1a	0	0.66389	0.6282	1
Li	1a	0.75816	0.18785	0.63612	1
V	1a	0	0.32723	0.13363	1
V	1a	0.5	0.67277	0.63363	1
O	1a	0	0.33539	0.5	1
O	1a	0.5	0.34827	0.53386	1
O	1a	0.26554	0.80539	0.54434	1
O	1a	0.73446	0.80539	0.54434	1
O	1a	0.23446	0.19461	0.04434	1
O	1a	0	0.65173	0.03386	1
O	1a	0.76554	0.19461	0.04434	1
O	1a	0.5	0.66461	0	1

a = 6.3276 Å, b = 5.4487 Å, c = 4.9498 Å, $\alpha = \beta = \gamma$ = 90°

References

1. T. Kondo, *et al.*, *Chem. Mater.,* 2024, **36,** 2495-2507.
2. F. Izumi and K. Momma, *Solid State Phenom.*, 2007, **130,** 15-20.
3. T. Roisnel and J. Rodríquez-Carvajal, *Mater. Sci. Forum*, 2001, **378-381,** 118-123.
4. K. Momma and F. Izumi, *J. Appl. Crystallogr.*, 2011, **44,** 1272-1276.
5. P. Giannozzi, *et al.*, *J. Phys. Condens. Matter*, 2009, **21,** 395502.
6. P. Giannozzi, *et al.*, *J. Phys. Condens. Matter*, 2017, **29,** 423003.
7. E. Iwama, *et al.*, *ACS Nano* 2016, **10,** 5398-5404.
8. W. Weppner and R. A. Huggins, *J. Electrochem. Soc.*, **124,** 1569-1578.
